# Patients’ Experiences, Expectations, Motivations, and Perspectives around Urinary Tract Infection Care in General Practice: A Qualitative Interview Study

**DOI:** 10.3390/antibiotics12020241

**Published:** 2023-01-24

**Authors:** Stefan Cox, Maud Vleeming, Wesley Giorgi, Geert-Jan Dinant, Jochen Cals, Eefje de Bont

**Affiliations:** Department of Family Medicine, Maastricht University, 6229 HA Maastricht, The Netherlands

**Keywords:** general practice, urinary tract infections, antimicrobial stewardship, qualitative research

## Abstract

While there are many alternatives to antibiotics for the symptomatic treatment of urinary tract infections (UTIs), their application in practice is limited. Among other things, general practitioners (GPs) often feel pressure from patients to prescribe antibiotics. To gain a better understanding of why this happens and where this pressure originates from, we investigated experiences, expectations, motivations, and perspectives of patients with UTIs in general practice. During this qualitative study we performed 14 semi-structured online interviews among female UTI patients in general practice. Interviews were based on a topic list derived from sensitising concepts. All the interviews were recorded, transcribed, and analysed using a constant comparative technique. Three main categories emerged from the data; (1) experienced versus unexperienced patients with UTI, (2) patient’s lack of knowledge, and (3) patients feeling understood. Inexperienced patients consult a general practitioner for both diagnosis and symptom relief, while experienced patients seem to consult specifically to obtain antibiotics. In addition, patients have a lack of knowledge with regard to the diagnosis, treatment, self-care, and cause of UTIs. Finally, patients’ satisfaction is increased by involving them more in the process of decision making, so they feel understood and taken seriously. Patients’ expectations in UTI management in general practice often arise during their first experience(s) and play a major role in subsequent episodes. In conclusion, preventing misconceptions is especially important in the inexperienced patient group, as this may prevent future overtreatment of UTIs. In addition, involving patients in the decision making process will lead to greater understanding of the GP’s treatment choices.

## 1. Introduction

Almost half of all women will experience a urinary tract infection (UTI) during their lifetime. To obtain antibiotic treatment, patients in the Netherlands need a prescription supplied by a medical doctor. It is therefore unsurprising that UTIs are the most common reason for women to contact a general practitioner (GP) [1,2]. Besides antibiotics, GPs can also apply a “wait-and-see” approach with analgesic medication to treat UTIs in otherwise healthy women. This is possible due to the self-limiting character of most UTIs [3]. The overprescribing of antibiotics can lead to antibiotic resistance, which limits treatment options for future infections [4] and lead to side effects in many patients, such as nausea and diarrhea [5]. Previous research has shown that women are willing to avoid antibiotics because of this [6]. Despite these findings, antibiotics are still widely used in daily practice [7]. Strikingly, while the Dutch national guidelines on UTIs in general practice have been adapted to incorporate the restrictive use of antibiotics, GPs do not always follow the guidelines, especially in out-of-hours general practice care [8].

GPs cite time pressure, unfamiliarity with current guidelines, and applied pressure by patients as reasons to prescribe in cases where this is perhaps not indicated. Despite the available knowledge on the (un)necessary treatment of UTIs and the risk of overprescribing of antibiotics, the implementation of that knowledge in daily practice leaves much to be desired [8]. Therefore, we wanted to gain better insight into what patients expect from their GP and what influences their decision to contact a GP. This insight could subsequently aid in adapting and improving UTI care to the needs of the patient. The primary aim of this study was therefore to investigate the experiences, expectations, motivations, and perspectives of patients with UTIs in general practice.

## 2. Results

Ultimately, 92 patients completed the short questionnaire. We invited 14 patients for an interview, including patients of varying age, experience, and education level (Table 1). Three main categories emerged from the data, distributed over different topics. Each category is discussed in detail below. Table 2 shows representative interview quotes exemplifying the topics and themes discussed in the result section proper.

### 2.1. Experienced versus Unexperienced Patients

The first notable theme we found in the interviews was that there was a difference in several areas between patients with UTI experience (≥3 episodes in their lifetime) and patients who were inexperienced (<3 episodes) with UTIs. Remarkably, experienced patients cited atypical symptoms, such as a shivering sensation during voiding, more often than inexperienced patients did. Additionally, we saw a difference in motivation for patients to contact their GP. Inexperienced patients contacted their GP in order to understand what was causing their symptoms and how to get rid of them. However, they often chose to consult other sources first, such as family, friends, or the internet, therefore they often already had a suspicion of having a UTI when contacting the GP.

Notably, experienced patients indicated that they recognised the complaints from previous episodes and that they were convinced that the complaints constituted a UTI. However, there was a dichotomy in the moment at which they contacted their GP. Some of the experienced patients indicated that they would contact their GP as soon as they recognised their complaints as a UTI, with the purpose of starting treatment as soon as possible. Others in the experienced group indicated that they consciously wait before making contact despite recognition of the symptoms. Patients mentioned two reasons for this: on the one hand, patients indicated that they already know the forthcoming advice to first wait and try some self-care treatment, which they had usually already started themselves. On the other hand, there were patients who indicated that their urine generally does not confirm a UTI in the initial phase, and that they therefore consciously wait for the development of more severe symptoms in order to increase the likelihood of a positive test at the moment of consultation.

Furthermore, the care request with which patients decide to contact their GP are different. Inexperienced patients often indicated that they contacted their GP because they were uncertain about the underlying cause of their symptoms. They are expecting a definite diagnosis when they make contact, as well as a solution to their symptoms. Experienced patients are often looking for an explanation as to why they suffer from UTIs so often and highly value further examination by the GP or even a referral to the hospital. Furthermore, experienced patients indicated that they know that the GP and the GP’s assistant assign great importance to the diagnostic tests, while the patients themselves see the tests more as an obstacle to overcome before obtaining treatment. As mentioned previously, some purposefully try to exacerbate their symptoms to increase the chance of a positive urine test. Nonetheless, all patients see it as a necessary step, since patients understand that GP’s need something objective, such as diagnostic tests, to base their decision on.

### 2.2. Patient’s Lack of Knowledge

The second theme that emerged was patients’ lack of knowledge. Misconceptions exist about the diagnosis, treatment, self-care advice, and the cause of UTIs. This lack of knowledge is both conscious and unconscious. Some patients were confident about their knowledge of certain aspects of UTIs, but when we consider scientific literature and guidelines, their confidence seems to be misplaced.

The level of knowledge with regard to self-care methods varied among patients, even among experienced patients. Many patients indicated that they received little self-care advice from their GP, but that they obtained most of the advice on self-care methods from family or friends, or that they found it on the internet. Because the source of the information is not the GP in these cases, patients often question the reliability of the information. Still, it differs from patient to patient as to whether they believe that providing information on self-help advice is required.

There is a striking difference between patients with regards to taking pain medication. Part of the patients that considered pain the most disturbing complaint during UTI did not take pain medication. These patients indicated that they had not considered paracetamol or ibuprofen, since they thought they were more suitable for other types of pain, such as headaches.

Additionally, many patients indicated that they did not know how their GP chose the type of antibiotic treatment, while they would greatly appreciate an explanation. Some patients indicated that due to this lack of clarity, they had made assumptions about how the GP arrives at the choice of the treatment themselves. One of these assumptions was that the GPs could be financially incentivised in their choice of antibiotic, and that they choose certain antibiotics because it benefits the GPs themselves.

There is also little knowledge about the adverse effects of antibiotics. Some patients indicated that they were aware of antibiotic resistance, but most interviewees were unfamiliar with the concept. It is striking that patients generally have apprehensive thoughts about antibiotics and medication (preferably as little as possible, not good for my body, etc.), but that they also indicated that they contacted the GP with the specific aim of obtaining an antibiotic prescription. While most patients knew that antibiotics also have negative effects, almost none were able to mention specific negatives of antibiotic use.

### 2.3. Patients Feeling Understood

Patients who were satisfied with primary care contact indicated that this is mainly because they had the idea that the caregiver, be it the GP or the GP’s assistant, listened to them and took them seriously.

The feeling of being listened to increases by including the patient in decisions regarding disease management. Patients would like to see more shared decision making in several areas. Patients most frequently mentioned that they would appreciate contributing to the choice of treatment. They indicated that they do not always understand the choice of treatment and would like their previous experiences (e.g., with certain antibiotics) to be included in the final decision. One patient suggested that shared decision-making would lead to increased therapy adherence.

In addition, some patients indicated that they are certainly open to discussing differing opinions with their GP, but whenever this is the case, they would like to talk it over and devise a treatment plan together. This is not the case for all patients, since some indicated that they feel that the doctor has the expertise and should have the final say.

Inexperienced patients also indicated that it is important that healthcare providers realise that while UTIs are commonplace in their practice, the situation can be foreign for the patient. Lastly, patients feel they lack guidance in practical matters (how to collect a urine sample, where and when to hand in a urine sample, how results are reported, etc.)

## 3. Discussion

### 3.1. Summary

The expectations, motivations, and perspectives of patients with urinary symptoms visiting the GP are based on previous experiences with the condition, causing experienced patients to value antibiotic treatment much more highly than diagnostics compared to inexperienced patients. Furthermore, patients, irrespective of experience, seem to lack knowledge about antibiotic resistance and the self-limiting nature of UTIs. Ultimately, patients highly value the opinion of their GP on management decisions, as long as it is backed by a proper explanation.

### 3.2. Strengths and Limitations

In qualitative interviews there is always a risk of socially desirable answers. To combat this, we allowed participants to be in the familiar environment of their own choosing, made possible by the online nature of the interviews. Additionally, we tried to elucidate the underlying meaning of opinions and experiences, but there is a risk of misinterpretation, despite various applied methods such as member checks, paraphrasing, and investigator triangulation. All researchers had a medical background with an interest in general practice and infections, which might have influenced their views and interpretation of the data.

For some patients, UTI episodes that we discussed had occurred many years ago, which could be a source of recall bias. However, we think that retrospective interviews were the most pragmatic method to collect the data we were interested in, namely patients’ opinions and how these opinions were formed.

This research focused on the Dutch health care system. How generalizable the research is for other countries depends on the similarity of organization of the healthcare system in those countries. However, we believe that the results are generalizable to most Western countries.

### 3.3. Comparison with Existing Literature

Previous studies on UTIs investigated alternatives to antibiotics, such as a wait-and-see approach, self-care measures and/or delayed antibiotic prescriptions. The majority of women included in those studies indicated that they were willing to undergo alternative treatment [6,9,10,11,12]. Despite these findings, we learned during the interviews that the demand for antibiotics among patients is high, especially in experienced patients. However, further questioning revealed that the aim of the patients was not necessarily to obtain antibiotics, but rather a reduction in symptoms, which corroborates what has been found in previous studies [6,13]. Patients’ preference for antibiotics arises from previous positive experiences [14], a reason mentioned frequently in our study. Therefore, we hypothesise that due to the previous treatments, patients link symptom reduction to antibiotics and consider them necessary to cure UTIs, a notion held by patients from previous studies [14,15,16]. This association needs to be broken in order for antibiotic prescribing for UTIs to change [17].

In order to achieve this, GPs should see or speak to women with urinary symptoms more often during a (telephone) consultation, especially if it is their first episode. Women with urinary symptoms rarely see their GP in the Netherlands, since most UTI contacts are handled by the GP’s assistant. This sometimes causes women to feel that their complaints are unheard. If women would be able to express their concerns and expectations to the GP, GPs would be able to take into account those concerns and expectations while offering treatment. Inexperienced women want an explanation for and an alleviation of their symptoms, while experienced patients want symptom relief and further examination into the cause of their recurrent infections. Inexperienced patients’ needs are borne from worry and fear of the unknown symptoms, while the need of experienced patients is a result of frustration with the recurrent symptoms [18].

Similar reasons for visiting the GP were found in respiratory tract infection (RTI) patients [19,20,21]. In the past, RTI patients’ expectations for antibiotic treatment was higher than today [22]. This can be attributed to successful campaigns against antibiotic use for RTI, as well as the increased inclusion of patients in the decision making process regarding their treatment [20]. Furthermore, information tools such as pamphlets were highly valued by both patients and GPs for providing information about the usually benign course of RTIs, reducing the need for antibiotic prescriptions [21]. Since the UTI patients in our study also mention a lack of knowledge about UTIs and their causes, it might be prudent to implement something similar for UTI care. Previous studies have shown some success in reducing antibiotic prescribing for UTIs when using information leaflets [23] or shared decision making [24,25,26]. A focus of any such leaflets should be on the potential side effects of antibiotics, since in our study as well as previous studies it has been shown that the patient population is unaware of them [17].

When patients consult their GP with symptoms that they believe to be a UTI and the GP subsequently confirms this suspicion, the patient will always consider those symptoms to constitute a UTI. This is of course a logical way of thinking. However, the danger here is that when the initial episode is incorrectly diagnosed as a UTI, the patient will make an erroneous association between their symptoms and having a UTI. This could explain why (primarily experienced) patients often cite atypical symptoms in our study. A complicating fact in this case is that the diagnostic tests currently available to GPs are either inaccurate (urinary dipstick) or time consuming (urinary culture) [27]. An accurate point-of-care test would make it much easier for GPs to correctly diagnose patient symptoms and could prevent overtreatment with antibiotics in patients with urinary symptoms and undertreatment in patients with any other conditions that might be at play [15,28,29].

Strikingly, a previous study has shown that 57% of the patients with a UTI consider pain to be the main complaint, but that only 29% actually use pain medications [30]. Similarly, we found that some of the patients in our study did not know that they could use pain medication against the pain associated with a UTI. In a previous study we showed that more knowledgeable patients were willing to delay antibiotic use [31]. Therefore, we think there is an important role for GPs and their assistants in informing patients about the (often) self-limiting course of UTIs and the use of pain medication to alleviate the symptoms associated with the infection and thereby reduce UTI-associated antibiotic prescriptions. If patients are made aware of how “harmless” a UTI usually is, they will probably consult less often or consult without the immediate expectation of antibiotics.

### 3.4. Implications for Practice

Patients develop expectations around UTI care based on previous episodes of urinary symptoms. GPs should remind themselves of this when dealing with inexperienced patients that they are consulting because of urinary symptoms, since the treatment used for the first episode sets a precedent for any future episodes. In addition, GPs should be aware of the importance of explaining the self-limiting course of UTIs, thereby not only battling antibiotic resistance but also decreasing their own future workloads by demedicalizing UTI management. Furthermore, if GPs explain why a certain therapy is chosen, patients will be more understanding of the choice of treatment and will feel that their concerns have been taken seriously.

## 4. Materials and Methods

We performed a thematic analysis based on naturalistic inquiry, done through semi-structured interviews with patients who received care in general practice or during out-of-hours general practice because of urinary symptoms. During the interviews we enquired about patients’ experiences, expectations, motivations, and perspectives around UTI care in general practice. During the reporting of the outcomes of the study, we used the Consolidated Criteria for Reporting Qualitative Research (COREQ): a 32-item checklist [32].

This study focused on all UTI care in which the GP is involved in the Netherlands. During office hours, anyone in the Netherlands with urinary symptoms can contact their own GP. Otherwise, patients can contact the out-of-hours general practice centre. When contacting the out-of-hours centre, patients always speak with a triage nurse first, who determines the severity of symptoms and whether a GP consultation is necessary.

Recruitment for and the conducting of the interviews took place from November 2021 to February 2022. Recruitment took place via social media (Facebook, Instagram, LinkedIn) and posters placed in general practices, supermarkets, gyms, and at the university, therefore none of the participants had a prior relationship with the researchers. Women interested in participating were able to register voluntarily by filling out a short online questionnaire inquiring about age, education level, frequency of UTIs, and the type of primary care contacted for urinary symptoms (GP out-of-hours care, GP practice, GP assistant). Women who had a UTI in the past year and who had contacted a general practice or out-of-hours general practice centre were included. Exclusion criteria were known urinary tract abnormalities, pregnancy and/or an indwelling catheter at the time of the UTI. All patients received written information and provided informed consent prior to participation. The obtained data were anonymised. The Medical Ethics committee of the Maastricht University Medical Centre in Maastricht approved this study (2019-1294). We used purposive sampling based on age, education level, and frequency of UTIs. We categorized educational level into primary education, secondary education, intermediate vocational education, higher vocational education, and university. Frequency of UTIs was categorised into “<3 UTI episodes” and “≥3 UTI episodes”. The categories for UTI frequency were based on the Dutch College of Family Practitioners (NHG) guidelines and their definition for recurrent UTIs [3]. The categories for other characteristics were based on the scientific literature [10,33].

We developed a topic list prior to the interviews in which a number of key questions and subjects were prepared. This topic list was iteratively adapted between interviews based on the data obtained through the use of sensitizing concepts [34]. The questions were based on existing literature and previous experiences with qualitative research [8]. We asked about patients’ experiences, expectations, motivations, and perspectives around the care of UTIs in general practice. We conducted two practice interviews to test the topic list and to train the interviewer. An example of the final version of the topic list is included as Appendix A.

One female and two male interviewers (MV (medical student), WG (medical student), and SC (PhD candidate)) conducted each of the online semi-structured interviews, which lasted between 25 and 40 min. MV was the main interviewer, while WG and SC asked additional questions to obtain additional perspectives and to elucidate any confusion. Since the interviews took place online via Zoom meetings (Zoom Video Communications, Inc., San Jose, CA, USA), patients were in a familiar environment and the interviews could continue despite the COVID-19 pandemic. Interviews were both video and audio recorded. After 14 interviews we decided against conducting additional interviews, since we had already reached our goal of purposeful sampling and no new topics had emerged in the last interviews (data saturation). Transcripts of the interviews were not returned to the interviewees.

We used the constant comparative technique to analyse the data. SC and MV performed inductive content analyses using open coding, axial coding, and selective coding schemes using NVivo 12 Pro (QSR International Pty Ltd., Burlington, MA, USA). Inconsistencies about coding were discussed with JC (professor of General Practice) and EdB (general practitioner) until a consensus was reached.

## 5. Conclusions

Patients’ experience with UTI care influences their expectations, motivations, and perspectives around UTI care in general practice. Inexperienced patients expect to be diagnosed and to receive an explanation for their symptoms. They are motivated to contact the GP by their ignorance for what is ailing them, as well as the severity of their symptoms or the debilitation that they cause. Their perspective on UTIs is mostly one of initial panic, since they are unaware of what causes their symptoms and what they can do to alleviate them. Experienced patients expect to be treated with antibiotics, since treatment with antibiotics was what alleviated the symptoms during their previous episodes (at least according to them). Furthermore, they expect further examination to find the root of the problem, since many feel frustrated by their recurrent UTIs. Their motivation to contact the GP stems from this frustration as well as wanting to be rid of the symptoms. Their perspective on UTI care is also rooted in frustration, since they feel that they have to overcome many hurdles to obtain their preferred treatment.

Regardless of experience, almost all patients lack knowledge about UTIs. For some aspects, such as how the GP arrives at their treatment of choice, or how to treat themselves, patients consciously experience this ignorance. For other aspects, such as the drawbacks of antibiotics or the use of NSAIDs for the treatment of UTIs, this lack of knowledge is not stated by the patients themselves, but can be inferred from the given answers. Furthermore, all patients feel the need to be taken seriously by the health professional they are in contact with, be it the doctor’s assistant or the GP themselves.

All in all, GPs should consider the patient’s experience with UTI when they present with urinary symptoms, since patients’ expectations change with increased experience. Furthermore, more attention should be given to patients who present with urinary symptoms for the first time, since the diagnosis attached to the symptoms will be called upon by the patient in future episodes. However, patients with urinary symptoms should be invited for a consult more often than happens now in the Netherlands in order to make this possible. This would provide GPs with the opportunity to educate their patients about the self-limiting course of UTIs and the drawbacks of antibiotics, which in turn might cause GPs to perceive less pressure to prescribe antibiotics.

## Figures and Tables

**Table 1:** Characteristics of the participating patients based on the questionnaire (*n* = 14).

Interview	Age (Years)	Education	Lifetime Frequency of UTI
1	40	University	≥3 times
2	23	Intermediate vocational education	≥3 times
3	37	Intermediate vocational education	≥3 times
4	78	Secondary education	≥3 times
5	37	University	≥3 times
6	28	Intermediate vocational education	≥3 times
7	36	Higher vocational education	<3 times
8	32	University	≥3 times
9	30	Higher vocational education	<3 times
10	57	Intermediate vocational education	<3 times
11	69	University	<3 times
12	54	Intermediate vocational education	<3 times
13	23	University	<3 times
14	51	University	<3 times

**Table 2:** Themes that originated from the data, ordered by theme and topic.

Theme	Topic	Interview	Quote
Experienced versus inexperienced patients	Atypical symptoms of experienced patients	4	Patient: “Well I don’t actually feel anything, I just feel a kind of shiver when I have to pee. I don’t have to go more often, don’t feel any pressure, also don’t feel any pain. Only a strange feeling when I have to pee….”
	Moment of contacting GP for inexperienced patients, motivation for contacting for inexperienced patients I	14	Patient: “First I started Googling that Sunday; like what’s actually wrong with me. Quite quickly, I got the idea: I think this is an acute bladder infection. And then I immediately called the GP on Monday.” Interviewer: “Were the complaints the primary reason for you to call, or was it more that you knew that it was a cystitis that made you get in touch with the GP?” Patient: “The complaints. I thought: “I have to walk my dog, I have to do groceries, I have get something to get off the couch again.”
	Moment of contacting GP for experienced patients I, motivation for contacting for experienced patients I	13	Patient: “I actually knew that I simply had a bladder infection and needed a course of antibiotics.” Interviewer: “How did you know?” Patient: “I recognised the symptoms from 3 years ago.”
	Moment of contacting GP for experienced patients II, motivation for contacting for experienced patients I	1	Patient: “I know that it [the dipstick test] will turn out positive once there is blood in my urine. So that’s what I wait for.” … “And I know that when I hand this [my urine] in I’ll get my antibiotics. In fact, sometimes I use that [knowledge] by drinking less and by putting more pressure on my bladder when I pee.”
	Inexperienced patient on UTI diagnostics, motivation for contacting for inexperienced patients II	7	Patient: “I’m not a doctor or GP, so I need them to diagnose, I am unable to do that myself. So if he deems it [the diagnostic test] necessary then I just do it.” Interviewer: “… Was getting the diagnosis the most important reason for you to consult?” Patient: “Well, I just really wanted to get rid of the symptoms.”
	Experienced patient on UTI diagnostics	1	Interviewer: “You already indicated yourself that you only visit [the GP] when you think that the urine will turn out positive. Is your urine always tested when you consult your practice?” Patient: “Always! Even if I’m crying at the counter begging that it’s absolutely unnecessary and that I’m definitely sure [that I have a bladder infection]. Without it [handing in urine], I will never ever get antibiotics.” Interviewer: “How do you feel about that?” Patient: “I think it’s a good development. It’s a huge difference compared to 20 years ago, you can tell that a lot has really changed there, in general I think. It is also different with animals now, I have a lot of pets and in the past you got antibiotics for everything and were allowed to powder and pulverize it yourself, but here I can tell that people understand that it’s not the way to go about it.”
Patients’ lack of knowledge	Self-help advice I, antibiotics I	14	Patient: “In any case, more information on what I can do myself. What could alleviate it [the symptoms]? Does the medication actually help immediately, or could you wait for a day? So more information.”
	UTI in general, self-help advice II	14	Interviewer: “And did you receive any additional explanation about your bladder infection?” Patient: “No.” Interviewer: “Would that be something you want? Tips, advice, an explanation?” Patient: “No, I wouldn’t. However, there were a lot of people in my environment that offered me advice. Like cranberries, but I didn’t take them seriously.” … “because people also just get their tips from somewhere, their grandmother, or on Facebook... However, I am genuinely curious about cranberries.”
	Self-help advice III	12	Interviewer: “Just now we discussed cranberry juice, of which you doubt whether it’s effective. Where would you look for this information?” Patient: “Nowadays you start on the internet of course, Google, very straightforward. But when you get into it a bit you’ll find many different opinions, therefore my second place to look for information is still the GP.”
	Pain medication I	14	Patient: “It’s a different type of symptom, I use them [painkillers] for headaches or neck pain, but not for urinary tract pain. That’s not something that comes to mind.”
	Pain medication II	5	Patient: “I maybe use paracetamol once a year.” Interviewer: “Also during a bladder infection?” Patient: “No, because I don’t feel any pain in that case.”
	Antibiotics II	5	Patient: “They [the GP or the pharmacist] don’t tell you anything but you still have to pay for it every time. And then I get the idea, they are just like: madam has had this antibiotic for a year now, it has almost been a year so we can’t charge 6 euros for that so we just give something else for now, that’s the idea I have. That’s probably not the case, it’s probably that this antibiotic works on this bacterium, but they don’t tell you that.”
	Antibiotics III	10	Patient: “The side effects; you know that on the third or fourth day, that maybe differs from patient to patient, that your stool possibly might come out a little faster due to the antibiotics, that it becomes a little thinner. That is what I’ve come to expect from an antibiotic course, then I just imagine that my body is cleansed in the process as well.”
Patients feeling understood	Difference in perception of disease severity I	7	Patient: “I liked that they took me so seriously even though they probably had something like it’s just a bladder infection, ma’am don’t go like that, you’re not dying. But in my eyes, because I’ve never had it [a UTI] before, I thought what happened here, so I liked that I was taken so seriously.”
	Influence on treatment choice	4	Interviewer: “Was there a difference between the antibiotics you got?” Patient: “Last time it was a sachet with one dose, the Furabid I have to take for a week.” Interviewer: “Which do you prefer?” Patient: “The sachet with a single dose.” Interviewer: “And why do you prefer that one?” Patient: “Because you don’t have to think about it anymore….”
	Importance of diagnostics	10	Interviewer: “Suppose that the next time you visit a GP the doctor says: ‘You don’t have the typical pain or burning sensation during urination, I am in doubt whether this is a bladder infection.’ What would be your reaction?” Patient: “Then I’ll ask if they would check my urine anyway.” Interviewer: “So the test has a lot of diagnostic value for you?” Patient: “Yes, … I’m not going to walk around with questions. It’s either ‘yes’ or ‘no’ and ‘you have to do this’ or ‘you have to do that’, but not ‘just figure it out’.”
	Difference in perception of disease severity II	9	Patient: “I did think that I was given rather little information about ‘how’ and ‘what’, but I do understand it. It is a very common complaint in women of course, so I also think that they thought something like: ‘oh this is normal and this is a very common problem.”

## Data Availability

The data presented in this study are available on request from the corresponding author. The data are not publicly available to ensure participants’ privacy.

## Appendix A

**Interview topic list**

Main question:

What are the motivations, expectations, and experiences of patients consulting a general practitioner (GP) due to a possible urinary tract infection (UTI)?

Subquestions:

Questions about the period before consultation:

Situation at home

◦ What do patients do at home before they contact their GP?

Motivation and moment of calling

◦ Why do patients decide to call their GP?
◦ When do patients decide to call their GP?

Triage

◦ What do patients think of triage by telephone?
◦ Who is able to estimate the severity of the symptoms most accurately?
◦ Are patients open to the advice of triagists?
◦ Do patients follow the advice of triagists or is only a doctor able to convince them?
◦ Do patients visit the GP against the advice of the triagist? Why?

Questions on the period of consultation:

Expectations of the consultation

◦ What do patients expect from their GP during consultation?
◦ Do patients dare to ask specifically what they want to know?

Experience

◦ How do patients experience a consultation at the GP?
◦ Do the advice or the actions of the GP scare the patients?
◦ What do patients think of the fact that a different doctor will see them at the out-of-hours centre?

Information

◦ What information do patients obtain from their GP during consultation?
◦ What information do patients miss?
◦ What information would patients find important to obtain when they experience a possible UTI?

Questions on the period after consulting at the out-of-hours general practice center:

Satisfaction

◦ When are patients satisfied?
◦ What causes patients to be satisfied with the consultation?
◦ What reassures patients?

Motivation for re-consultation

◦ What causes patients to re-consult?

Integration of previous experiences/advice during later episodes

◦ Do patients apply information obtained during a possible future UTI?
◦ Does this influence the choice to consult earlier/later in a later episode?

Points of improvement

◦ How can UTI consults be improved according to patients?
